# Cognitive Alterations in Old Mice Are Associated with Intestinal Barrier Dysfunction and Induced Toll-like Receptor 2 and 4 Signaling in Different Brain Regions

**DOI:** 10.3390/cells12172153

**Published:** 2023-08-27

**Authors:** Annette Brandt, Franziska Kromm, Angélica Hernández-Arriaga, Inés Martínez Sánchez, Haktan Övül Bozkir, Raphaela Staltner, Anja Baumann, Amélia Camarinha-Silva, Rochellys Diaz Heijtz, Ina Bergheim

**Affiliations:** 1 Department of Nutritional Sciences, Molecular Nutritional Science, University of Vienna, 1090 Vienna, Austria; 2 Animal Nutrition Department, Institute of Animal Science, University of Hohenheim, 70593 Stuttgart, Germany; 3 Department of Neuroscience, Karolinska Institute, Biomedicum, 17177 Stockholm, Sweden

**Keywords:** hippocampus, pathogen-associated molecular patterns, cognition, intestinal barrier, prefrontal cortex, aging

## Abstract

Emerging evidence implicate the ‘microbiota–gut–brain axis’ in cognitive aging and neuroinflammation; however, underlying mechanisms still remain to be elucidated. Here, we assessed if potential alterations in intestinal barrier function and microbiota composition as well as levels of two key pattern-recognition receptors namely Toll-like receptor (TLR) 2 and TLR4, in blood and different brain regions, and depending signaling cascades are paralleling aging associated alterations of cognition in healthy aging mice. Cognitive function was assessed in the Y-maze and intestinal and brain tissue and blood were collected in young (4 months old) and old (24 months old) male C57BL/6 mice to determine intestinal microbiota composition by Illumina amplicon sequencing, the concentration of TLR2 and TLR4 ligands in plasma and brain tissue as well as to determine markers of intestinal barrier function, senescence and TLR2 and TLR4 signaling. Cognitive function was significantly impaired in old mice. Also, in old mice, intestinal microbiota composition was significantly altered, while the relative abundance of Gram-negative or Gram-positive bacteria in the small and large intestines at different ages was not altered. Moreover, intestinal barrier function was impaired in small intestine of old mice, and the levels of TLR2 and TLR4 ligands were also significantly higher in both portal and peripheral blood. Furthermore, levels of TLR2 and TLR4 ligands, and downstream markers of TLR signaling were higher in the hippocampal and prefrontal cortex of old mice compared to young animals. Taken together, our results suggest that even in ‘healthy’ aging, cognitive function is impaired in mice going along with an increased intestinal translocation of TLR ligands and alterations of TLR signaling in several brain regions.

## 1. Introduction

While overall life expectancies increase, healthy life-span, meaning the average number of years that an individual is expected to live in a state of self-rated good or very good health, based on current mortality rates and prevalence of good or very good health, is not increasing at a same rate [1]. Indeed, in 2020, life expectancy in the EU at birth was estimated to be 83.2 and 77.5 years for women and men, respectively, whereas the number of healthy life years at birth was thought to be 64.5 years for women and 63.5 years for men, representing ~77.5% and 81.9%, respectively, of the total life expectancy [2,3]. Studies have also suggested that aging increases the odds of developing various diseases including cancer, metabolic diseases like type 2 diabetes and metabolic dysfunction-associated steatotic liver disease but also diseases related to cognitive function like Alzheimer’s disease (AD) and dementia [4,5]. For the latter, it has been projected that in Europe, dementia cases will increase from 7.7 million in 2001 to 15.9 million cases by 2040 [6], with prevalence ranging from 6.86 to 9.06% among the 65+ population in various European countries in 2018 [6]. Despite these alarming numbers and the enormous costs associated [6], universally accepted prevention and treatment strategies are still lacking as molecular mechanisms underlying the development of aging associated cognitive impairments are still not fully understood.

In recent years, alterations of intestinal microbiota composition and microbiota associated metabolites were discussed to affect cognitive function [7]. Indeed, the so called ‘gut–brain axis’ has been shown to act as a bidirectional communication network between the central and the enteric nervous system [8]. Several tissues and organs including the brain, intestine and glands, as well as immune cells and intestinal microbiota, have been proposed to be critical herein (for an overview, see [8,9]). The results of several studies further suggest that the intestinal microbiota, be it directly through an activation of the vagus nerve or the activation of immune cells and an induction of the synthesis and release of neurotransmitters (for an overview, see [10]) or through metabolites derived from it [11] might influence the nervous system. For instance, both increases and decreases in prevalence and relative proportion of Bacteroides have been found in patients with dementia and AD [12,13,14]. Furthermore, it has been shown that a transfer of fecal microbiota of overweight subjects showing cognitive impairments in learning, immediate as well as short-delayed recall, and working memory to healthy lean mice results in cognitive impairments in recipient mice. The latter findings have been related to changes in microbial metabolism of one-carbon and aromatic amino acids [15,16]. The results of several studies also suggest that the development of AD is associated with a higher susceptibility to microbial pathogen-associated molecular patterns (PAMPs) neurotoxicity [17,18]. Herein, an increase in Toll-like receptor 2 (TLR2) ligands like zymosan and lipoteichoic acids has been shown to induce neurodegeneration in mice, which was found to be related to their microglial-mediated toxicity [19]. For TLR4 activation, data are conflicting (for an overview, also see [20]). Indeed, in some studies, early stimulation of TLR4 (and TLR2) signaling was shown to improve AD-associated learning and memory impairments in rats [21]. In contrast, in other studies, an activation of TLR4 through lipopolysaccharide (LPS) exacerbated neurodegeneration and inflammation, while a deletion of TLR4 improved cognitive dysfunction in aging mice was associated with alterations of blood–brain barrier integrity and inflammatory processes [22,23]. In line with these findings, a developmental TLR4 deficiency seems to improve memory retention and spatial reference memory acquisition in mice [24]. Here, the expression of TLR4 in microglia seems to contribute mainly to the cognitive changes [25,26,27]. However, how levels of TLR2 and TLR4 ligands and their signaling pathways are altered in the brain in the natural course of aging and if this is related to the development of cognitive impairments and changes of intestinal microbiota and barrier function has not yet been assessed.

Starting from this background, the present study aimed to determine if (1) the natural course of ‘healthy’ aging is associated with changes in intestinal microbiota composition and barrier function, and subsequently, increased levels of TLR2 and TLR4 ligands in blood and brain as well as an activation of depending signaling cascades in different brain regions, and if (2) these alterations are paralleling changes in aging related cognitive dysfunction.

## 2. Materials and Methods

### 2.1. Animals

Animals were handled in accordance with the European Convention for the Protection of Vertebrate Animals used for Experimental and Other Scientific Purposes and housed in a specific pathogen free (SPF) barrier facility accredited by the Association for Assessment and Accreditation of Laboratory Animal Care. The local institutional animal care and use committee approved all the experiments. Male C57BL/6J (JAX:000664) 4-month-old (=young mice) and 24-month-old (=old mice) mice (n = 8, except for analysis carried out in brain tissue and retrobulbar blood where samples of n = 2 mice per group were lost due to technical issues unless otherwise stated. Also, see figure legends) were bred and housed in groups under standard SPF controlled conditions and had free access to food (standard pellet food #V1534-300, Ssniff Spezialdiäten GmbH, Soest, Germany) and tap water. Experiments for both age groups were run in parallel and terminated at the same time. Animals were anesthetized with a mixture of ketamine/xylazine (100 mg ketamine/kg body weight; 16 mg xylazine/kg body weight, i.p. injection) and killed by cervical dislocation. Blood was collected in heparinized tubes from retrobulbar and portal vein and centrifuged at 4000 rcf, 12 °C for 8 min to obtain plasma. Brain sections of hippocampus (HIP) and prefrontal cortex (PFC) as well as intestinal tissue samples from proximal small intestine (‘small intestine’) and large intestine were collected and snap-frozen or fixed in neutral-buffered formalin. Parts of proximal small and large intestinal tissue were used to build everted gut tissue sacs as detailed below.

### 2.2. Working Memory in a Spatial Novelty Preference Y-Maze Paradigm

Two weeks before, sacrifice behavioral tests were performed (see Figure S1), as previously described by others, to assess short-term spatial memory [28]. In brief, a single mouse was placed in the Y-Maze, in which two of the three arms were open. During this ‘sample phase’ of 5 min, the mouse was allowed to explore two (‘start arm’ and ‘familiar arm’) out of the three arms. The animal was then placed back in a cage for 1 min while the Y-maze was cleaned. The respective mouse was then again introduced to the maze with now three open arms for 2 min (choice phase). To assess the working memory, the % of time the mouse spent in the third, ‘new arm’ as well as the time the animal spent in the ‘familiar’ as well as in the ‘start’ arm was determined. Studies have shown that mice have a natural tendency to explore novel surroundings more than familiar ones [29]. SMART Video Tracking System V3.0 (Panlab Harvard Apparatus, Barcelona, Spain) was used to analyze the videos. Moreover, the total distance moved and total arm visits during the choice phase was recorded and analyzed in order to assess general locomotor activity. One mouse in each group tried to escape the maze and was, therefore, excluded from the analysis.

### 2.3. Everted Gut Sac and Xylose Permeation Measurement

Pieces of small and large intestinal tissue, respectively, were dissected from the mice and everted with a rod as described in detail before [30]. Sacs were filled with 1× Krebs–Henseleit-bicarbonate buffer containing 0.2% (*w*/*v*) bovine serum albumin (KRH buffer) and incubated for 5 min in gassed KRH buffer containing 0.1% (*w*/*v*) D-xylose (Merck KGaA, Darmstadt, Germany) ex vivo. Detection of xylose permeation was assessed as described previously by assessing xylose concentration within the sacs [31,32] and calculated in µmol/cm. Due to technical problems when building the everted tissue sacs, it was only possible to analyze n = 6 in large intestinal and in 4-month-old small intestinal tissue sacs.

### 2.4. Histological Evaluation of Brain and Intestinal Sections

The right hemisphere of the brain and small and large intestine were embedded in paraffin and sections (4 µm, large intestinal sections n = 6/group due to technical issues) were stained with hematoxylin (1 min, Hematoxylin Solution, Gill No. 2, 4 g/L, Sigma Aldrich, Darmstadt, Germany) and eosin (2 sec, Eosin Y solution, alcoholic, 0.5% (*w*/*v*) in acidified ethanol, Sigma-Aldrich, Germany). To assess villous atrophy in small intestine, villus height and crypt depth was assessed while crypt depth was also assessed in large intestine using an analysis system (Leica Applications Suite X, Leica, Wetzlar, Germany) integrated in a microscope (Leica DM6 B, Leica, Germany). Neutrophil granulocytes were stained in 4 µm brain sections using Naphthol AS-D Chloroacetate Kit (Sigma-Aldrich, Germany) as described by the manufacturer. For the detection of Nissl bodies in the cytoplasm of neurons, 4 µm brain sections were deparaffinized and hydrated in ethanol. After staining for 10 min in 0.1% cresyl violet solution at 37 °C (diluted in distilled water and 0.3% (*w*/*w*) glacial acetic acid) sections were washed and differentiated in 95% ethanol, dehydrated, cleared and mounted.

### 2.5. Measurement of Ligands of TLR2 and TLR4

Concentration of TLR2 and 4 ligands in portal vein and retrobulbar plasma of mice were assessed using a commercially available SEAP reporter HEK293 cell assay (Invivogen, San Diego, CA, USA) as detailed previously [33]. Pieces of snap frozen HIP and PFC, respectively, were homogenized in pyrogen-free water, treated with ultrasound for 1 min and supernatant was collected after centrifugation. After determining total protein content with employing a commercially available kit based on the methods of Bradford (Bio-Rad Laboratories, Hercules, CA, USA), supernatant was used in SEAP reporter HEK 293 cell assay as well, and data were presented normalized to protein in mg/mL. As protein levels were too low in one sample of PFC of young mice, only n = 5 were analyzed. In brief, transfected HEK293 cells were challenged with plasma or tissue homogenate, respectively, for 16 h and color change of medium was assessed, being indicative of ligand concentration, at 655 nm.

### 2.6. RNA Isolation and Real-Time qPCR

RNA was extracted from both HIP and PFC using Trizol (TRItidy G™, AppliChem, Darmstadt, Germany) according to the instructions of the manufacturer. In brief, after homogenization in TRItidy G and adding chloroform, samples were centrifuged and RNA was precipitated with isopropanol from upper, aqueous phase and RNA pellet was washed with 70% ethanol 2 times. Pellets were dissolved in DEPC H_2_O at 55 °C for 10 min. After determination of RNA concentration at 260 nm and a DNase digestions (RQ1 Rnase-Free Dnase #M6101, Promega GmbH, Madison, WI, USA), all but one sample of HIP (4 months old) showing too low RNA concentration were transcript to cDNA (reverse transcription system #A3500 obtained from Promega GmbH, USA) following the instructions of the manufacturer. The amplification of genes listed in Table S1 was performed with real-time polymerase chain reaction (PCR) as described in detail before, normalized to 18S and using the comparative C_T_-method [34,35]. In brief, iTaq™ Universal SYBR^®^ Green Supermix (Bio-Rad Laboratories, Hercules, CA, USA) was used to prepare PCR mastermix and primers were added in a final concentration of 3 pmol/µL. Using the CFX Connect cycler (Bio-Rad Laboratories, USA), PCR was carried out following the following temperature protocol with an initial step at 95 °C for 30 s followed by 40 cycles of a 2-step PCR (95 °C for 5 s, 64 °C for 30 s (for 18S: 55 °C)) and a final melting curve analysis.

### 2.7. ELISA

Concentrations of intestinal-fatty acid binding protein (I-FABP) and plasminogen activator inhibitor-1 (PAI-1) in plasma of mice (n = 7 due to lack of sample) was assessed with commercially available ELISAs according to the manufacturer’s description (mouse I-FABP ELISA, #NBP2-82214, Novus Biological, a Biotechne brand, Minneapolis, MN, USA; mouse PAI-1 DuoSet ELISA, #DY3828, Biotechne, USA). According to the instructions of the manufacturer (Biotechne, USA), for the determination of PAI-1 protein, a 96-well-plate was coated with 4 µg/mL of capture PAI-1 antibody, incubated overnight at 25 °C and washed with washing buffer (0.05% Tween20 in PBS). After blocking wells with 1% bovine serum albumin (BSA) diluted in PBS for 1 h at 25 °C, plasma samples (diluted 1:30 in 1% BSA/PBS, random order) and PAI-1 standards (one well per sample) were added and incubated for 2 h (25 °C). Followed by a washing step, wells were incubated with the detection antibody (400 ng/mL, 2 h, 25 °C) and Strepatvidin-HRP (20 min, 25 °C). After a final washing step, substrate solution (1:1 mixture of H_2_O_2_ and Tetramethylbenzidine) was added for 20 min and reaction was stopped with 2N H_2_SO_4_. Extinction was measured at 450 nm (wavelength correction: 540 nm) and PAI-1 concentration was calculated applying the PAI-1 standard curve ranging from 600 pg/mL to 0 pg/mL. Following the instructions of the commercially available I-FABP ELISA (Novus Biological, a Biotechne brand, USA), plasma samples (diluted 1:15 in Sample Diluent, random order) and I-FABP standard (ranging from 2.5 ng/mL to 0 ng/mL) (one well per sample) were added to each well and incubated for 90 min at 37 °C. After an incubation with the biotinylated detection antibody (1 h, 37 °C) and a washing step, HRP conjugate was added for 30 min at 37 °C. After a final washing step, 90 µL of substrate reagent was added followed by 50 µL of stop solution per well. Extinction was measured at 450 nm and I-FABP concentration in samples was calculated by using the standard curve.

### 2.8. Western Blot

To determine markers of inflammation, 20 µg (2 µg/µL) (or 2 µg (0.2 µg/µL) for albumin) of total plasma protein were loaded in each pocket on a 12% polyacrylamide gel and transferred to a polyvinylidene difluoride membrane (Bio-Rad Laboratories, USA) and protein levels of albumin, cluster of differentiation 14 (CD14), C-reactive protein (CRP), Growth/differentiation factor-15 (GDF-15) and p16 were determined as detailed before [36,37]. In brief, after blocking for 1 h at room temperature with gentle shaking, membranes were incubated with the primary antibodies (albumin, Cell signaling, Danvers, MA, USA; CDKN2A/p16, Biorbyt, UK; CD14, Santa Cruz, Dallas, TX, USA; CRP, Santa Cruz, USA; GDF-15, Santa Cruz, USA; list of antibodies used and respective dilutions see also Table S2) at 4 °C overnight with gentle shaking followed by a washing step with 1× Tris-buffered saline with Tween20 (TBST, 3 × 10 min) and incubation with the respective secondary antibody for 1.5 h shaking at room temperature (detailed information on antibodies used please also see Table S2). After a second washing step with 1 × TBST (3 × 10 min), bands were detected using Super Signal West Dura kit (Thermo Fisher Scientific, Waltham, MA, USA). Densitometric analysis of bands were performed using ImageLab Software V 6.1.0 (Bio-Rad, USA) and normalized to albumin.

### 2.9. Immunohistochemical Staining

Immunohistochemical staining of paraffin embedded small and large intestinal tissue sections (4 µm large intestinal sections n = 6/group due to technical issues) to determine occludin (Thermo Fisher Scientific, USA) and zonula occludens-1 (ZO-1) (Thermo Fisher Scientific, USA) protein was performed as previously described in detail [38]. In brief, sections were deparaffinized and rehydrated in ROTI^®^Histol (Carl Roth GmbH & CoKG, Karlsruhe, Germany) followed by different ethanol solutions (100%, 95%, 70%, distilled H_2_O, 10 min, respectively) and an incubation with protease (2 mg/mL, 10 min, 37 °C) as well as a washing step in 1 × PBS for 2 × 5 min. Following an overnight incubation at 4 °C with the primary antibodies (details see Table S2), slides were washed again and incubated with respective peroxidase-linked secondary antibodies (see Table S2) for 30 min at room temperature. After a final washing step, sections were incubated with a so-called Peroxidase Envision Kit (Agilent Dako, Santa Clara, CA, USA), followed by a counterstaining with hematoxylin (Hematoxylin Solution, Gill No. 2, Sigma Aldrich, Germany), and a rehydration in the above mentioned ethanol solutions and ROTI^®^Histol. Sections were covered with entellan^®^ (Sigma Aldrich, Germany) and a cover glass. To detect TLR2 (proteintech, Rosemont, IL, USA) and TLR4 (abcam, Cambridge, UK) as well as ionized calcium-binding adapter molecule 1 (IBA1) (proteintech, USA) protein in paraffin-embedded brain sections (4 µm) of the right hemisphere, sections were deparaffinized and rehydrated as detailed above, demasked with heat-induced epitope retrieval and Tris EDTA buffer (10 mM Tris base, 1 mM EDTA, 0.05% Tween20, pH9) (TLR2, IBA1) or 10 mM sodium citrate buffer (TLR4). For TLR2 and IBA1 detection, sections were blocked with peroxidase blocking solution for 10 min (Agilent Dako, USA). Sections were then either incubated in 1% BSA (*w*/*v*, in TBS) (TLR2, TLR4) or 5% goat serum in TBS (IBA1) for 1 h followed by 2 × 5 min washing steps (1 × TBST). After an overnight incubation with the respective primary antibodies at 4 °C (TLR4) or 1.5 h at room temperature (TLR2, IBA1) (for antibody details, see Table S2), sections were washed 2 × 5 min in 1 × TBST and incubated with peroxidase-linked secondary antibodies for 30 min at room temperature (for details, see Table S2) and antibody binding was detected by Peroxidase Envision Kit (Agilent Dako, USA), followed by a counterstaining with hematoxylin and covering as detailed above. The intensities of the occludin, ZO-1 and IBA1 protein staining of each microscopic field, respectively, were evaluated using an analysis system (Leica Applications Suite V4.5, Leica, Germany) integrated in a microscope (Leica DM6 B, Leica, Germany) as detailed before [38]. In brief, six (IBA1, 200× magnification) to eight (occludin, ZO-1, 400× magnification) pictures per section and mouse were analyzed and means were determined. The extent of the staining within the sections was defined as percent of the field area within the default color range.

### 2.10. Microbiota Analysis

Total DNA was extracted from rinsed small and large intestinal tissue samples using a modified Trizol protocol [39] (Trizol, Sigma Aldrich, Germany). DNA quality was determined by spectrophotometry. The region V1–V2 of the 16S rRNA gene was amplified using PrimeSTAR^®^ HS DNAPolymerase kit (TaKaRa, Beijing, China) as described previously [40]. Amplicons were purified and normalized using the SequalPrep Normalization Plate Kit (Thermo Fisher Scientific, USA). Samples were sequenced on an Illumina MiSeq platform using 250 bp paired-end sequencing chemistry. The Illumina demultiplexed paired-end sequences were processed using Qiime2 2019.10 [41]. Reads were quality filtered, error corrected, dereplicated and merged by the q2-dada2 plugin [42]. Taxonomy assignment of generated amplicon sequence variants (ASVs) was implemented in VSEARCH-based consensus and pre-fitted sklearn-based classifiers against the Silva SSU-rRNA database (v.138.1, 16S 99%; [43]). Unassigned sequences and the reads from organelles were removed. Bacteria were classified as Gram-positive or Gram-negative at the genus level with considering classified members. Primer-e version 6 [44] was used for the calculation of permutation analysis of variance (PERMANOVA) between sample groups and to calculate Shannon diversity index. A Wilcoxon test followed by BH procedure was conducted to analyze differences in the Shannon diversity index. MicrobiomeAnalyst [45] was used for further statistical analysis. Data were submitted to the European Nucleotide Archive under the accession number PRJEB55351.

### 2.11. Statistical Analysis

PRISM (Version 7.03, GraphPad Software, Inc., Boston, MA, USA) was used for statistical analysis. Outliers were determined with Grubb’s test before further statistical analysis. For each analysis, the normality of distribution was assessed. In case of normal distribution of data, an unpaired *t*-test was performed to determine statistical difference. The non-parametric Mann–Whitney test was used when data were not normally distributed, to determine statistical differences between groups. Statistical tests used and *p* values are reported in figure legends or Section 3. All data are presented as means ± standard error of means (SEM). *p* < 0.05 was defined to be significant.

## 3. Results

### 3.1. Effect of Aging on Markers of Cognitive Function and Senescence

**Figure 1:**
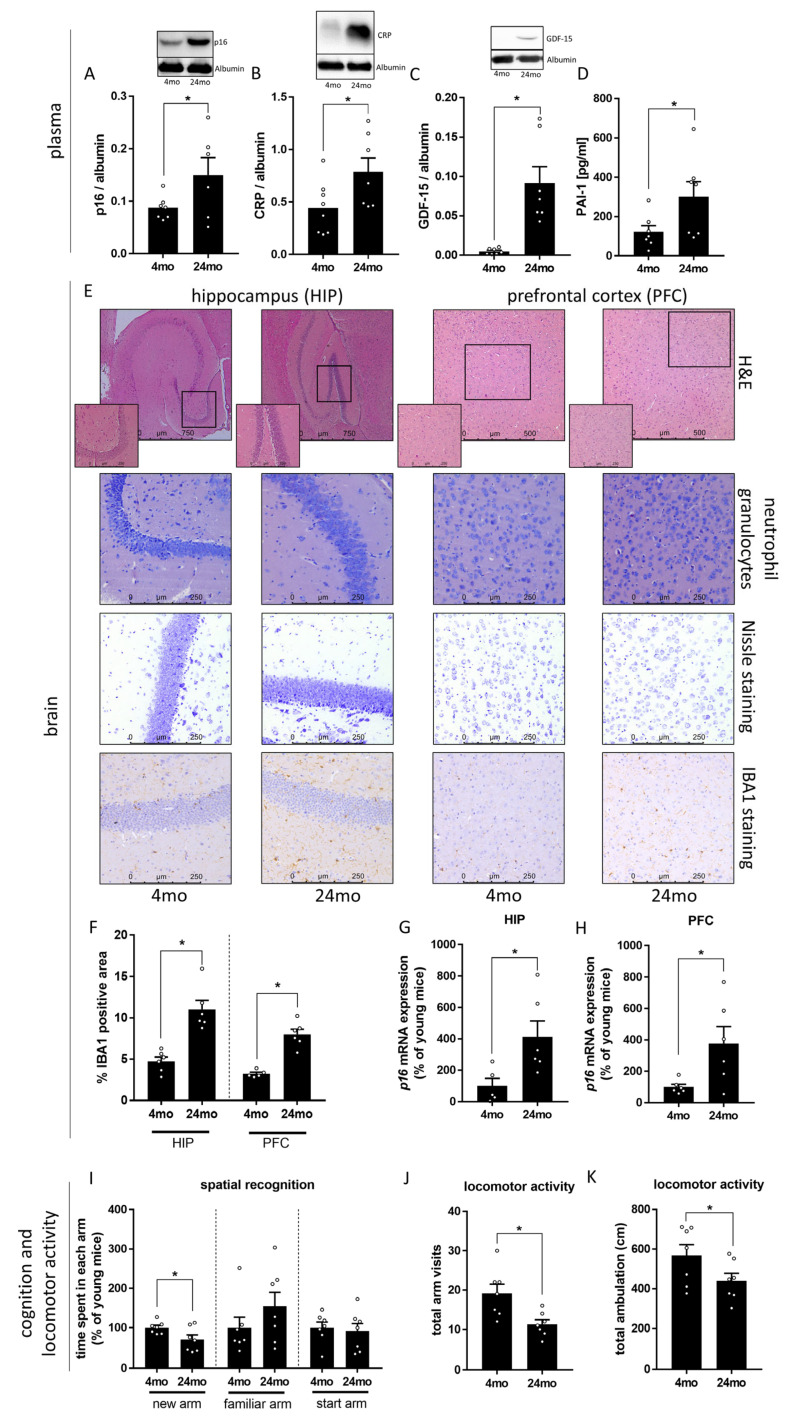
Effect of old age on markers of senescence and ‘inflammaging’ as well as cognition and locomotor activity in male mice. (**A**) p16 (24mo: n = 6 due to inadequate protein loading, unpaired *t*-test: *p* < 0.05), (**B**) C-reactive protein (CRP) (unpaired *t*-test: *p* < 0.05) and (**C**) Growth/differentiation factor-15 (GDF-15) (Mann–Whitney-test: *p* < 0.001) protein concentration in Western blots of plasma and (**D**) plasminogen activator inhibitor-1 (PAI-1) (unpaired *t*-test: *p* < 0.05) protein concentration in plasma. (**E**) Representative pictures of hematoxylin and eosin (H&E stained hippocampus (HIP, 50×, 200×) and prefrontal cortex (PFC, 100×, 200×), neutrophil granulocytes (200×), Nissle staining (200×) and of IBA1-staining (200×) in HIP and PFC. (**F**) IBA1-positive immunoreactive area (unpaired *t*-test: HIP: *p* < 0.001, PFC: *p* < 0.001) and (**G**,**H**) *p16* mRNA expression (unpaired *t*-test: HIP: *p* < 0.05, PFC: *p* < 0.05) in HIP and PFC. (**I**) Time spent in each arm (new arm: unpaired *t*-test: *p* < 0.05, familiar arm: Mann–Whitney-test: ns *p* > 0.05, start arm: unpaired *t*-test: ns *p* > 0.05), (**J**) total arm visits (unpaired *t*-test: *p* < 0.01) and (**K**) total ambulation (unpaired *t*-test: *p* < 0.05) in a spatial novelty preference Y-maze paradigm of 4-month-old (mo) and 24-month-old mice. Data are presented as means ± SEM, n = 5–8, see Section 2 for further details, * *p* < 0.05, ns = not significant.

As expected, concentrations of p16, CRP, GDF-15, and PAI-1, all described before as markers of senescence and so called ‘Inflammaging’ [46,47,48,49], were significantly higher in plasma of 24-month-old mice when compared to 4-month-old animals (Figure 1A–D). Although the gross histology of hippocampus and prefrontal cortex regions did not reveal any alterations (e.g., absence neutrophil infiltration), *p16* mRNA expression was significantly higher in both brain regions (Figure 1G,H). Furthermore, staining tissue section of HIP and PFC, respectively, for IBA1-positive immunoreactive areas (marker of microglia [50]) revealed significantly more positively stained areas in older mice suggesting that marker of microglia were increased in aging animals (Figure 1F).

Furthermore, short-term spatial memory was significantly lower in 24-month-old mice compared to 4-month-old animals, the 24-month-old mice spent significantly less time in the ‘new’ arm, whereas in the ‘start’ arm and ‘familiar’ arm, the residence times did not significantly differ between 4-month-old and 24-month-old mice. (Figure 1I). In addition, locomotor activity was significantly lower in 24-month-old mice compared to 4-month-old animals, as assessed by the total number of arm visits and total ambulation (cm) in the Y-maze test (Figure 1J,K).

### 3.2. Effect of Aging on Intestinal Microbiota Composition and Markers of Intestinal Barrier Function

**Figure 2:**
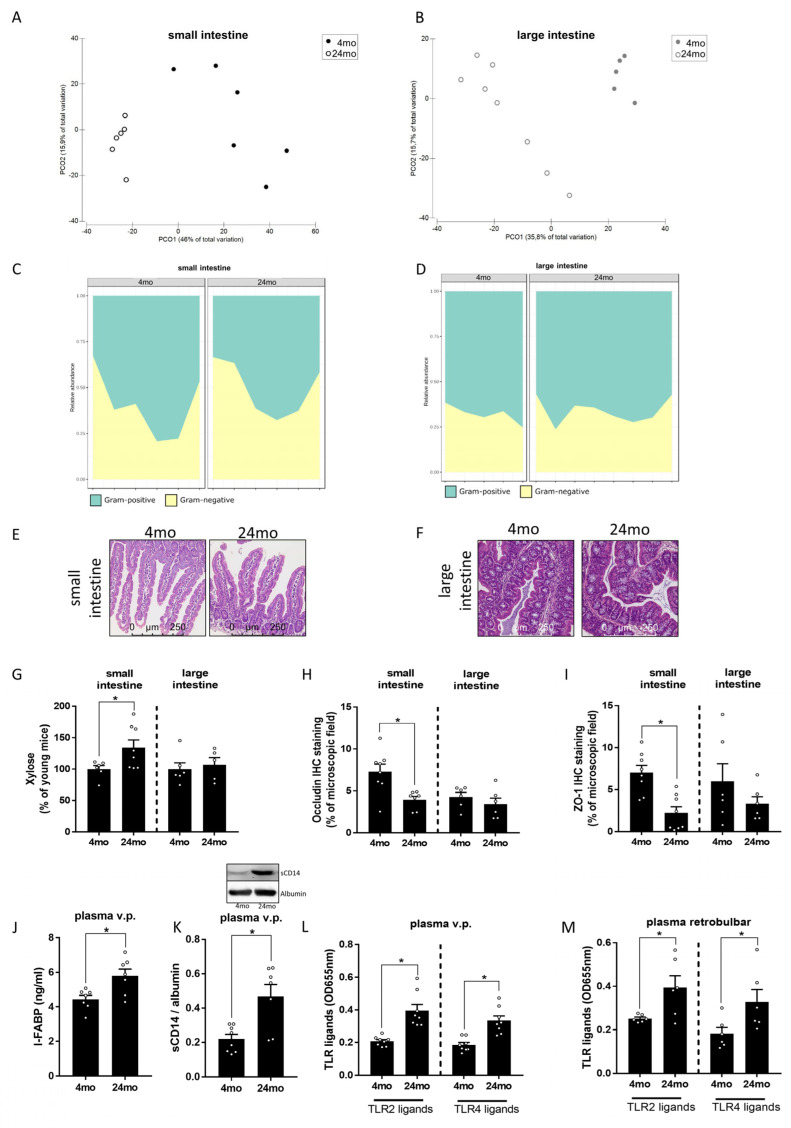
Effect of old age on microbiota composition and intestinal barrier in male mice. Principal Coordinate Analysis (PCoA) depicting the microbial communities of the samples from (**A**) small and (**B**) large intestine (each dot represents one sample) and area plot showing the abundances of Gram-positive and Gram-negative of the classified genera (samples are separated by age) of (**C**) small and (**D**) large intestine (x-axis showing individual samples). Representative pictures of hematoxylin and eosin stained (**E**) small intestine and (**F**) large intestine (200×), (**G**) xylose permeation (unpaired *t*-test: small intestine: *p* < 0.05, large intestine: ns *p* > 0.05) in everted gut sacs, densitometric analysis of (**H**) occludin (small intestine: Mann–Whitney-test: *p* < 0.01, large intestine: unpaired *t*-test: ns *p* > 0.05) and (**I**) zonula occludens-1 (ZO-1) (unpaired *t*-test: small intestine: *p* < 0.001, large intestine: ns *p* > 0.05) in immunohistochemical stained (IHC staining) small and large intestinal sections. (**J**) Intestinal-fatty acid binding protein (I-FABP) concentration (unpaired *t*-test: *p* < 0.01) in plasma and (**K**) cluster of differentiation 14 (CD14) protein concentration (unpaired *t*-test: *p* < 0.01) in Western blots of plasma, as well as ligands of Toll-like receptor 2 (TLR2) and TLR4 in (**L**) plasma from portal vein (v.p.) (unpaired *t*-test: TLR2 ligands: *p* < 0.001, TLR4 ligands: *p* < 0.001) and (**M**) retrobulbar plasma (unpaired *t*-test: TLR2 ligands: *p* < 0.05, TLR4 ligands: *p* < 0.05) of 4-month-old (mo) and 24-month-old mice. Data are presented as means ± SEM, n = 6–8, see Section for further details, * *p* < 0.05, ns = not significant.

To determine if the alterations found regarding markers of senescence and cognitive function were related to changes in intestinal microbiota composition and intestinal barrier function, microbiota composition was assessed in small and large intestines of young and old mice. We have shown before that intestinal microbiota composition differed between young and old mice [37,51]. In line with the previous findings of our group and others [37,51,52,53], the PERMANOVA revealed statistically significant differences in the total microbial communities by site (small intestine vs. large intestine, *p* < 0.001) and age (4 months vs. 24 months, *p* < 0.001) and the combination of both factors (*p* < 0.05) (Figure 2A,B). Alpha-diversity showed differences between all groups (*p* < 0.01) except between samples of the small intestine (*p* = 0.379) (Figure S2). As expected, the average abundance of Gram-positive vs. Gram-negative bacteria differed in small intestine and large intestines regardless of age groups. However, neither in small intestine nor in large intestine, a significant difference in relative abundance of Gram-negative bacteria were found when comparing young and old mice. Also, the relative abundance of Gram-positive bacteria did also not differ between age groups (Figure 2C,D). In line with our findings in brain tissue, the gross morphologies of the small and large intestines were similar between 4-month-old and 24-month-old mice (Figure 2E,F). Moreover, a marker of villous atrophy in the small intestine, like villus height and crypt depth and crypt depth in the large intestine, did not differ between 4-month-old and 24-month-old mice (Table 1). In contrast, intestinal permeability, as assessed ex vivo by xylose permeation, was significantly higher in small intestinal tissue of 24-month-old mice when compared to 4-month-old animals. Similar differences were not found in the large intestine (Figure 2G). Consistent with these findings, protein levels of the tight junction protein occludin and ZO-1 in small intestinal tissue were significantly lower in 24-month-old mice when compared to 4-month-old animals. Again, similar differences were not found in the large intestine (Figure 2H,I and Figure S3). Furthermore, despite the absence of differences in gross intestinal morphology between young and old mice, mean I-FABP protein levels, a protein considered as a marker of enterocyte damage but also having been shown to correlate with endotoxemia [54,55], in portal plasma were significantly higher in 24-month-old mice than in 4-month-old animals (Figure 2J). Similarly, concentrations of TLR2 and TLR4 ligands were significantly higher in both portal and peripheral plasma of 24-month-old mice compared to 4-month-old animals (Figure 2L,M). In line with these findings, a concentration of soluble CD14, which was shown to bind both TLR2 and TLR4 ligands [56,57], was also significantly higher in the peripheral plasma of 24-month-old mice compared to the younger animals (Figure 2K).

### 3.3. Effect of Aging on TLR2 and TLR4 Ligand Concentration as Well as on Markers of TLR2 and TLR4 Signaling in Prefrontal Cortex and Hippocampus

**Figure 3:**
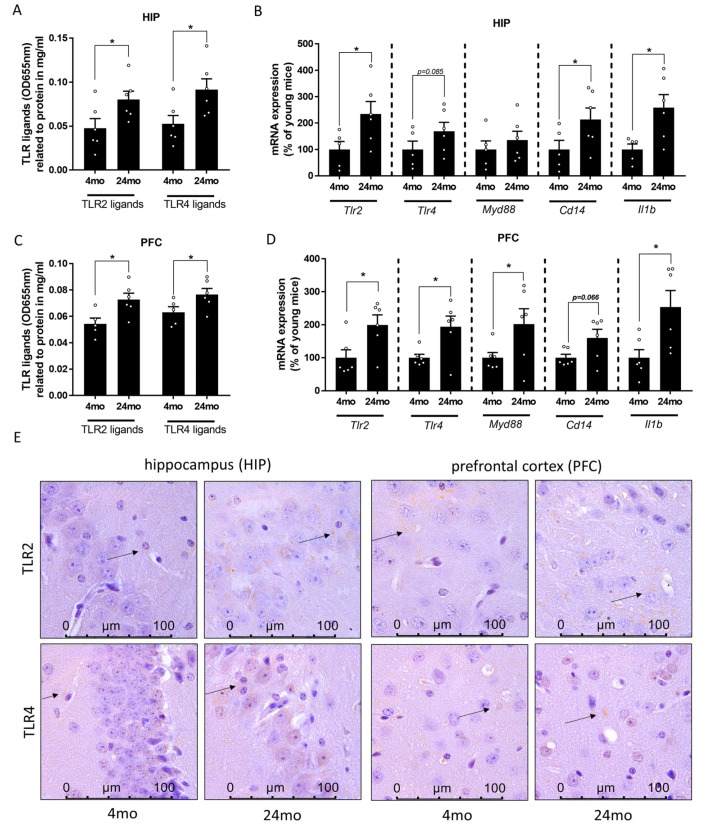
Effect of old age on TLR signaling cascade in brain in male mice. Ligands of Toll-like receptor 2 (TLR2) and TLR4 in (**A**) hippocampus (HIP) (unpaired *t*-test, TLR2 ligands: *p* < 0.05, TLR4 ligands: *p* < 0.05) and (**C**) prefrontal cortex (PFC) (unpaired *t*-test, TLR2 ligands: *p* < 0.05, TLR4 ligands: *p* < 0.05) and mRNA expression of *Tlr2*, *Tlr4*, *myeloid differentiation primary response 88* (*Myd88), cluster of differentiation 14 (Cd14)* and *interleukin 1b* (*Il1b)* in (**B**) HIP (unpaired *t*-test, *Tlr2*: *p* < 0.05, *Tlr4*: *p* = 0.085, *Myd88*: ns *p* > 0.05, *Cd14*: *p* < 0.05, *Il1b*: *p* < 0.05) and (**D**) PFC (*Tlr2*: Mann–Whitney-test: *p* < 0.05, *Tlr4*: unpaired *t*-test: *p* < 0.01, *Myd88*: Mann–Whitney-test: *p* < 0.05, *Cd14*: Mann–Whitney-test: *p* = 0.066, *Il1b*: unpaired *t*-test: *p* < 0.05). (**E**) Representative pictures of TLR2 and TLR4 staining (630×) in HIP and PFC (black arrows indicate an example of a positively stained area) of 4-month-old (mo) and 24-month-old mice. Data are presented as means ± SEM, n = 5–6, see Section 3 for further details, * *p* < 0.05, ns = not significant.

As the hippocampus has been shown to be the key brain area for spatial learning and memory [58], and the working memory seems to activate among others the prefrontal cortex [59], the TLR2 and TLR4 ligands as well as mRNA expression of *Tlr2* and *Tlr4* were then analyzed in the hippocampus and prefrontal cortex. Similar to the findings in portal and peripheral blood, concentrations of TLR2 and TLR4 ligands in tissue obtained from hippocampus and prefrontal cortex were significantly higher in 24-month-old mice when compared to 4-month-old animals (Figure 3A,C). In line with these findings, mRNA expressions of *Tlr2* and *Tlr4* were significantly higher in PFC of old age mice compared to young animals (Figure 3D). A similar pattern of expression was observed in the HIP, albeit only reaching statistical significance for *Tlr2* expression while *Tlr4* mRNA expression was also only higher by trend (*p* = 0.085) (Figure 3B). Similar differences were also seen when staining TLR2 and TLR4 protein in tissue section of hippocampus and prefrontal cortex (representative pictures in Figure 3E). Additionally, mRNA expression levels of *Cd14* and *interleukin 1b* (*Il1b*) were markedly higher in both HIP and PFC of old mice compared to young mice (*Cd14*: HIP *p* < 0.05, PFC *p* = 0.066; *Il1b*: HIP *p* < 0.05, PFC *p* < 0.05; Figure 3B,D). On the other hand, mRNA expression level of *myeloid differentiation primary response 88* (*Myd88*) was significantly higher in PFC, but not HIP (Figure 3B,D).

## 4. Discussion

Cognitive impairments in late life are frequent and may be related to the natural course of aging [60]. Still, studies have suggested that cognitive impairments might be a risk factor for the development of dementia and AD [61,62]. The number of individuals suffering from aging-associated cognitive alterations has been shown to increase rapidly [63]; however, molecular mechanisms underlying these alterations are still not fully understood. Similar to humans, several studies have shown that the natural course of aging in C57BL/6 mice seems also to be associated with memory and cognitive impairments [64,65,66,67]. Here, employing naïve 4 and 24-month-old C57BL/6 mice, we assessed if these “natural” healthy aging-associated cognitive alterations are related to changes in intestinal microbiota and barrier function, as well as TLR2 and TLR4 signaling in different brain regions. In line with previous findings from others and our group [37,48,68,69], in the present study, markers of senescence like p16, CRP, GDF-15, or PAI-1 were higher in the blood of old mice but also in the hippocampus and prefrontal cortex. Also, in line with the finding of others [50], the number of IBA1-positive cells was higher in both brain regions of older mice. These signs of senescence were paralleled with marked impairments of short-term spatial memory and locomotor activity. The latter findings are in line with those of others who have also shown that cognitive functions are impaired in the natural course of aging [64,65,66,67,70]. Taken together, our results and those of others suggest that aging, even in a healthy setting, seems to be associated with impairments in cognition.

### 4.1. Healthy Aging Related Cognitive Alterations Parallel Alterations of Intestinal Microbiota Composition and Barrier Function

In recent years, changes in intestinal microbiota and the so-called ‘gut–brain axis’ were discussed to be of crucial importance in the development of aging associated cognitive impairments (for an overview, see [71,72]). For instance, gnotobiotic mice inoculated with microbiota from aged mice were reported to suffer from impaired spatial and short-term memory [73]. Also, Boehme et al. recently demonstrated that a fecal microbiota transfer from healthy young mice to healthy old mice improves selective age-associated behavioral deficits of the latter [74]. In line with previous findings of our group and those of others [37,51,52,53] in the present study, intestinal microbiota composition differed significantly between age groups at the genus levels in both large and small intestine. However, relative abundances of Gram-positive and Gram-negative bacteria in small and large intestines were similar between age groups. Somewhat in line with these findings, the results of recent studies also suggested that intestinal microbiota, prior to shifts in composition, may react to host immune stimuli by changes of, e.g., immunomodulatory metabolite production [75]. If similar adaptive changes are also underlying the unaffected relative abundance of Gram-positive and Gram-negative bacteria in small intestine and large intestine found in the present study remains to be determined. Furthermore, in line with the findings of others and us in mice and humans [37,51,53,76], intestinal barrier function in small intestine as determined by assessing protein levels of tight junctions but also xylose permeation was significantly impaired in old aged mice. Furthermore, as TLR2 and TLR4 ligand concentration was higher in both, portal and peripheral blood of old aged mice, it could be that the clearance of bacterial toxins in liver was not sufficient. Interestingly, in the large intestine, neither xylose permeation nor tight junction protein levels differed between age groups. In contrast to the latter findings, Tran et al. reported before that in old-aged baboons, the intestinal barrier function is impaired in the large intestine [77]. The difference might be related to differences in species and methods employed to determine intestinal permeability (Ussing chamber vs. xylose permeation in everted sac in the present study). Further studies are needed to determine if the aging associated loss of intestinal barrier function is organ and/ or species specific. Still, the results of the present study add further weight to the hypothesis that changes in intestinal microbiota composition and barrier function leading to an increased translocation of TLR ligands are associated with the development of cognitive dysfunction in healthy aging. However, the contribution and impact of the alterations found at the level of the gut, e.g., regarding the relative abundance of specific bacterial strains as well as signaling cascades involved in the development of intestinal barrier dysfunction in settings of healthy aging remains to be determined. Also, the role of the mechanisms underlying the insufficient clearance of TLR ligands in the liver remains to be determined.

### 4.2. Healthy Aging Associated Cognitive Alterations Parallels with Alterations of TLR2 and TLR4 Signaling in Brain

The results of recent epidemiological and clinical studies suggest that the degree of cognitive alterations in elderly individuals is positively related to bacterial endotoxin and pro-inflammatory cytokine levels in plasma [14,78,79]. Also, several studies suggest that in humans with AD, levels of bacterial endotoxin as well as markers of TLR4 signaling are induced in hippocampus and neocortex or frontal cortex [80,81,82]. In the present study, in old mice with naturally evolved cognitive impairments, concentrations of TLR2 and TLR4 ligands, e.g., lipoteichoic acid and bacterial endotoxin were also higher in both brain regions studied when compared to young animals without cognitive impairments. Furthermore, the expressions of *Tlr2* and *Tlr4* and subsequent signaling molecules like *Myd88* and the proinflammatory cytokine *Il1b* were also higher in hippocampus and prefrontal cortex of old mice. However, there was no significant induction of *Myd88* mRNA expression in HIP of old mice, which might result from the fact that proteins such as MYD88 not solely be regulated by mRNA expression, but also post-transcriptionally be regulated by tyrosine phosphorylation [83]. Others assessing TLR2 and TLR4 expression in mice with AD also showed an increased expression and concentration of these TLRs in different brain regions [84,85]. Supporting the findings of the present study and further bolstering the hypothesis that a dysregulation of TLR signaling may be critical in the development of aging-associated cognitive alterations, results of ex vivo studies using organotypic hippocampal slice culture suggest that a TLR2 or TLR4 ligands activation of microglia may result in an enhanced NO release, and subsequently, network dysfunction [86,87]. Also, studies in aged TLR4 knockout mice suggest that cognitive function of these animals, when compared to wild-type mice, is superior, suggesting that a lack of TLR4 signaling might be of benefit [23,88]. Interestingly, and somewhat in contrast with the findings of the present study but also those of others, studies in aged TLR2 knockout mice suggest these mice suffer from a neurobehavioral dysfunction compared to age-matched wild-type animals [89]. Activated TLR signaling cascades might further trigger neuroinflammation, which is in line with the present findings, which suggest that 24-month-old mice show higher microglia activation associated with increased IBA1 expression in the brain [50].

Taken together, our results add further weight to the hypothesis that increased levels of TLR ligands, e.g., bacterial endotoxin, and lipoteichoic acids in brain tissue may be critical in the development of aging-associated cognitive alterations and inflammatory alterations in brain. Furthermore, our results do not preclude that other alterations associated with aging may also add to the naturally evolving cognitive alterations in old age. Moreover, others also have shown that not only intestinal barrier but also the blood-brain-barrier is altered in aged organisms [90], which might further contribute to the TLR-associated immune response in brain. However, the contribution of TLR2 and TLR4 ligands in brain tissue to the aging-associated cognitive impairments and molecular mechanisms involved remains to be determined.

### 4.3. Limitations

This study had several limitations which need to be taken into consideration when interpreting the data. Experiments in the present study were only performed in male mice, limiting the generalization to other sex and species. However, it has been shown before that both male and female mice suffer from cognitive impairments during aging [91,92,93], Also, microbiota composition has been reported to differ between young and old mice in both male and female [94]. Moreover, in the present study, cognitive alterations in aged mice was assessed by determining the working memory in a spatial novelty preference Y-maze paradigm, as described before [28], which might not reflect the full spectrum of cognitive decline during aging in mice [64,65,95,96]. Also, our study focused on describing alterations associated with aging. Therefore, if and how TLR ligands like the two shown to be elevated in the present study are involved in the development of cognitive impairments associated with ‘healthy’ aging remains to be shown.

## 5. Conclusions

In summary, the results of the present study further bolster the hypothesis that alterations of the ‘gut–brain axis’, and, herein, especially impairments of intestinal barrier function and an increased translocation of PAMPs are related with the natural course of healthy aging in male mice. Furthermore, these alterations parallel an induction of related signaling cascades in different brain regions and the development of aging-associated cognitive impairments. Our results also suggest that impairments found in intestinal barrier function and increases in TLR ligands in blood of an aging organism may also be reflective of changes in TLR ligands and signaling in the brain. However, molecular mechanisms underlying these alterations and their implications for the development of aging related cognitive dysfunction need to be determined in future studies. Also, whether alterations alike are also found in the brains of elderly humans and if this is related to impairments of cognitive well-being remains to be determined.

## Figures and Tables

**Table 1:** Effect of old age on marker of villous atrophy in small intestine and crypt depth in large intestine in male mice.

		4 Months	24 Months
small intestine	villus height (µm)	239 ± 14	241 ± 9.0
	crypt depth (µm)	94.4 ± 4.7	98.4 ± 7.3
large intestine	crypt depth (µm)	91.9 ± 1.8	89.0 ± 2.8

Data are presented as means ± SEM, n = 6–8, see Section 2 for further details. Unpaired *t*-test: villus height: ns *p* > 0.05, crypt depth (small intestine): ns *p* > 0.05, crypt depth (large intestine): ns *p* > 0.05. ns = not significant.

## Data Availability

Data will be made available on reasonable request. Sequencing data were submitted to the EuropeanNucleotide Archive under the accession number PRJEB55351.

## Supplementary Materials

The following supporting information can be downloaded at: https://www.mdpi.com/article/10.3390/cells12172153/s1, Figure S1: Study design. Figure S2: Effect of old age on (A) Shannon diversity of small intestine and large intestine in male mice and (B) taxonomy bar plot at genus level from small intestine and large intestine of young and old mice. Figure S3: Representative pictures (400×, 630×) of occludin and zonula occludens-1 staining in (A) small intestine and (B) large intestine; Table S1: Primer sequences; Table S2: Antibodies used.
