# Supplementary material for: Cognitive Alterations in Old Mice Are Associated with Intestinal Barrier Dysfunction and Induced Toll-like Receptor 2 and 4 Signaling in Different Brain Regions
Source: Cells. 2023 Aug 27;12(17):2153. doi: 10.3390/cells12172153 (PMC10486476; doi:10.3390/cells12172153)
Supplement: Supplementary file 1 [file cells-12-02153-s001.zip › cells-2491924-supplementary.pdf]

Supplemental Tables

Table S1. Primer sequences.

	Forward (5´-3´)	Reverse (5´-3´)
18S	GTA ACC CGT TGA ACC CCA TT	CCA TCC AAT CGG TAG TAG CG
Cd14	GAG TTG TGA CTG GCC CAG TCA GC	GCA AAA GCC AGA GTT CCT GAC
Il1b	TGG CTG TGG AGA AGC TGT GG	GTC CGA CAG CAC GAG GCT TT
Myd88	CCC TAG GGC AGA GGG GAA GA	ATG CCT GTG TGT GCA GAG GAG
p16	CCA AGA GCG GGG ACA TCA AG	AAG AAA AAG GCG GGC TGA GG
Tlr2	CTC CAC AAG CGG GAC TTC GT	GGC TCC AGC AAA ACA AGG A
Tlr4	AGC CAT TGC TGC CAA CAT CA	GCT GCC TCA GCA GGG ACT TC

Cd14: cluster of differentiation 14; Il: interleukin, Myd88: myeloid differentiation primary response 88; Tlr: Toll-like receptor.

Table S2. Antibodies used.

Application	primary antibody		secondary antibody	
	antibody, kDa	dilution/ diluted in	antibody	dilution/ diluted in
Western blot	Mouse anti- CD14 [sc-515785, Santa Cruz, USA], ~53kDa	1:500/ 5% skim milk powder	Horse anti- mouse IgG HRP-linked [7076, Cell signaling, USA]	1:5000/ 5% skim milk powder
	Mouse anti- CRP [sc-69770, Santa Cruz, USA], ~24kDa	1:500/ 5% skim milk powder	Horse anti- mouse IgG HRP-linked [7076, Cell signaling, USA]	1:5000/ 5% skim milk powder
	Mouse anti- GDF-15 [sc-515675, Santa Cruz, USA], ~50KDa	1:500/ 5% skim milk powder	Horse anti- mouse IgG HRP-linked [7076, Cell signaling, USA]	1:5000/ 5% skim milk powder
	Mouse anti- CDKN2A/p16 [orb378211, Biorbyt, UK], ~49kDa	1:500/ 5% BSA	Horse anti- mouse IgG HRP-linked [7076, Cell signaling, USA]	1:5000/ 5% BSA
	Rabbit anti- albumin [4929, Cell signaling, USA], ~67kDa	1:1000/ 5% skim milk powder	Goat anti- rabbit IgG HRP-linked [7074, Cell signaling, USA]	1:5000/ 5% skim milk powder
Application	primary antibody		secondary antibody	
	antibody	dilution/ diluted in	antibody	dilution/ diluted in
Immunohistochemical staining	Rabbit anti- Occludin [71-1500, Thermo Fisher Scientific, USA]	1:250/ 1x PBS	Anti-rabbit HRP labelled [K4003, Agilent, USA]	undiluted
	Rabbit anti- ZO-1 [61-7300, Thermo Fisher Scientific, USA]	1:100/ 1x PBS	Anti-rabbit HRP labelled [K4003, Agilent, USA]	undiluted
	Rabbit anti-TLR2 [17236-1-AP, proteintech, USA]	1:400/ 1% BSA in TBS	Anti-rabbit HRP labelled [K4003, Agilent, USA]	undiluted
	Mouse anti-TLR4 [ab22048, abcam, UK]	1:100/ 1% BSA in TBS	Anti-mouse HRP labelled [ab214879, abcam, UK]	undiluted
	Rabbit anti-IBA1 [10904-1-AP, proteintech, USA]	1:800/ 1x TBS	Anti-rabbit HRP labelled [K4003, Agilent, USA]	undiluted

BSA: bovine serum albumin, CD14: cluster of differentiation 14, CRP: C-reactive protein, GDF-15: Growth/differentiation factor-15, PBS: phosphate-buffered saline, TBS: Tris-buffered saline, TLR: toll-like receptor, ZO-1: zonula occludens-1.

Supplemental Figures

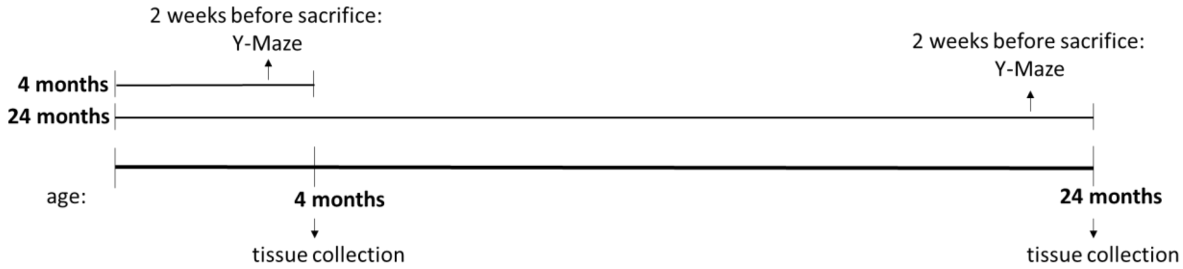

Figure S1: Study design.

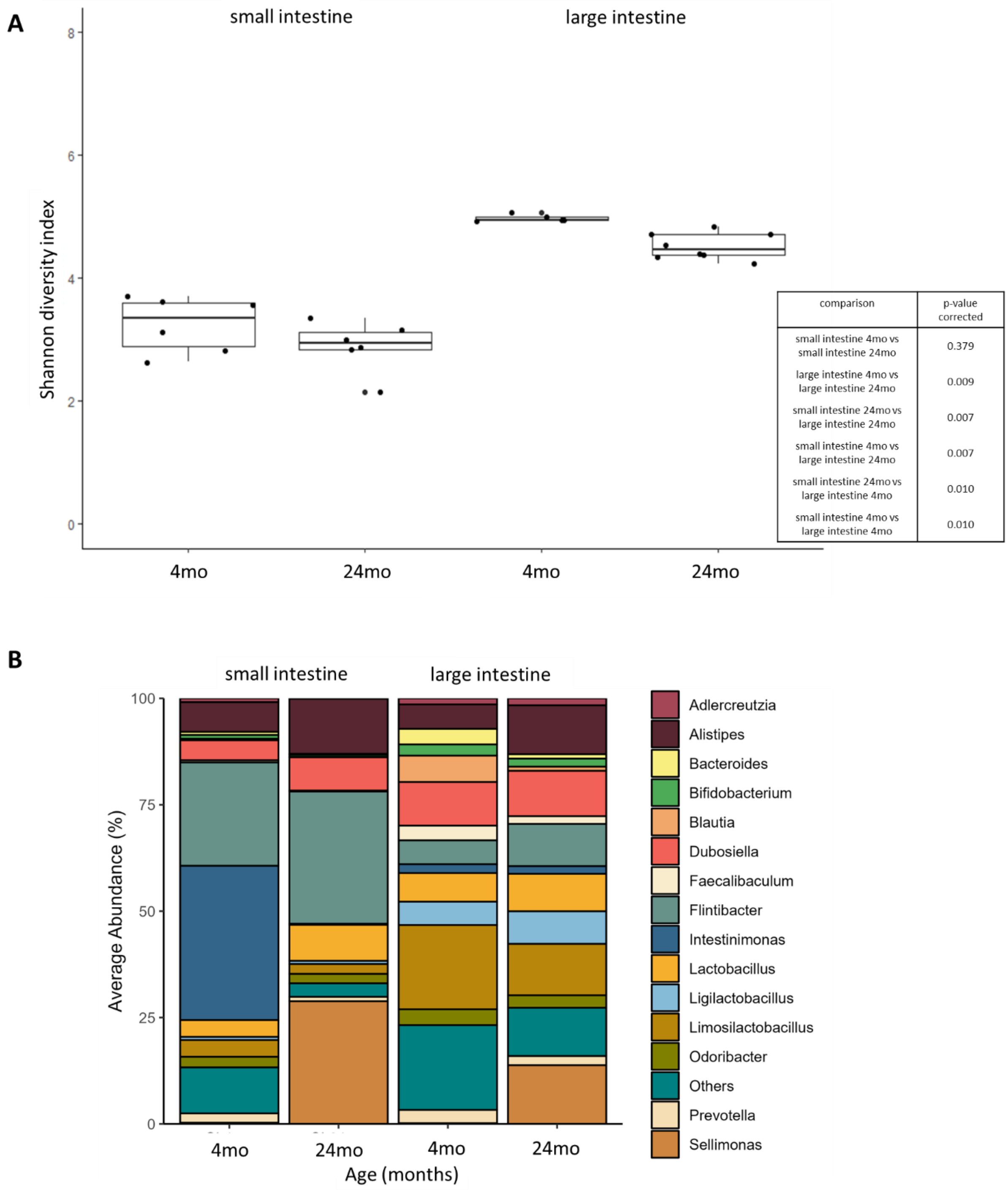

Figure S2: Effect of old age on (A) Shannon diversity of small intestine and large intestine in male mice and (B) taxonomy bar plot at genus level from small intestine and large intestine of young and old mice. mo-months.

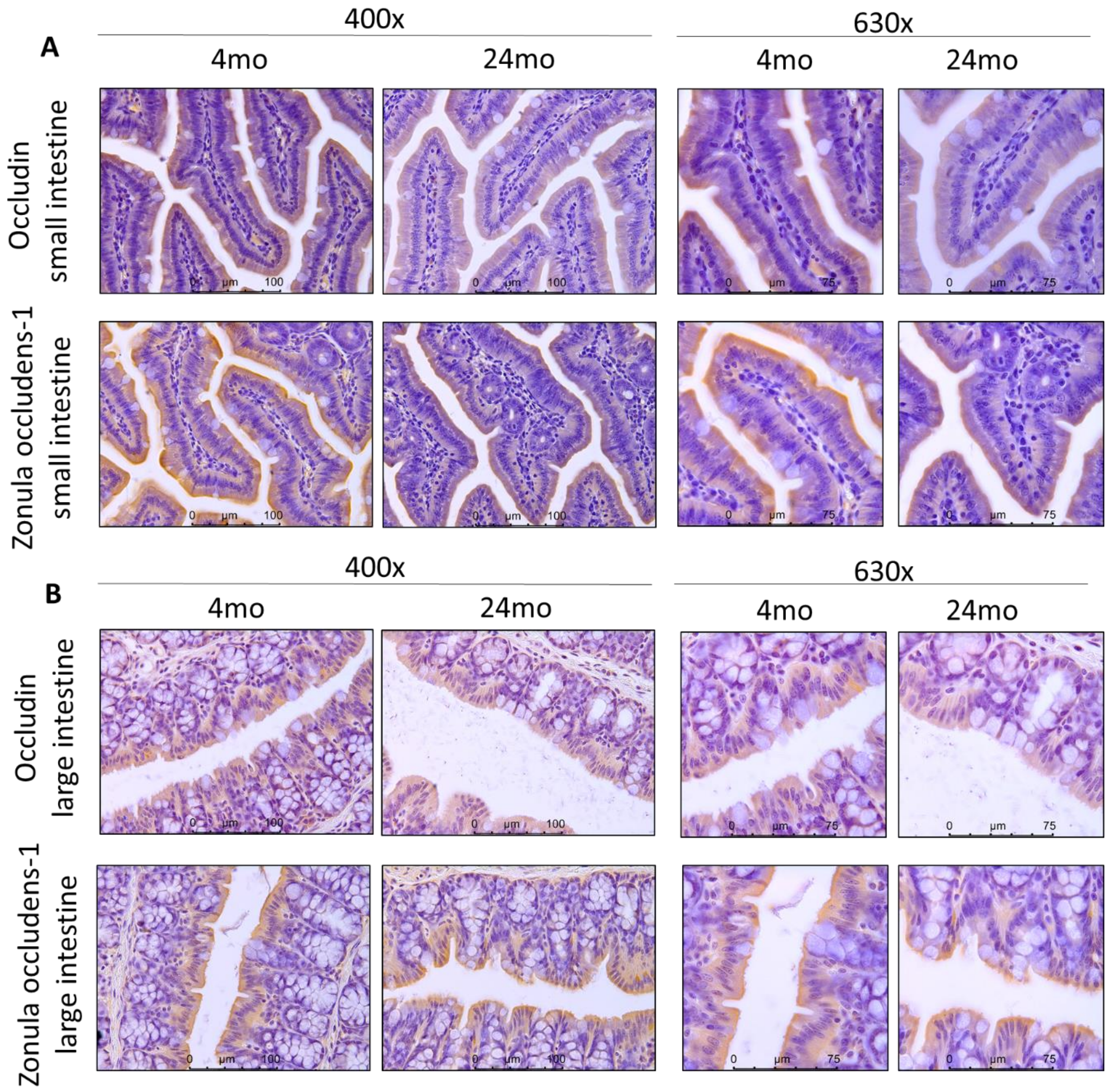

Figure S3: Representative pictures (400x, 630x) of occludin and zonula occludens-1 staining in (A) small intestine and (B) large intestine
